# Baths of Liebenzell

**Published:** 1831

**Authors:** 


### BATHS OF LIEBENZELL.

DR. BEATTIE, who accompanied our illustrious Sovereign and our amiable Queen, in three successive tours to the Continent, has published a journal of these tours, abounding in amusing anecdotes and interesting descriptions of the countries as well as the courts, where the Royal travellers sojourned, or through which they merely passed. The nature of the work did not permit our author to indulge much in mere medical matters, and therefore the contents of these volumes do not fall within the scope of our review. But, at this season of the year, when men and women are preparing to wing their flights to coast and country—to continents and isles, in search of health or pleasure, it may not be uninteresting to inform our readers—more especially for the benefit of their fair patients, that, in a certain part of the BLACK FOREST, there is a spring which has long been famous for specific virtues of a particular kind. From the days of Hubert the Hirch-hunter, down to the present epoch, the waters of LIEBENZELL have been the regeneration of many a sinking house—making the wilderness of domestic love to blossom like the rose—the vine to put forth its season — and mothers, who sorrowed once to sing for joy! But let the poet VATTERKENDER, tell the praises of LIEBENZELL.

If thy house be falling, falling—
No son his father's name to tell;
No bud, maternal bloom recalling—
*Send thy bride to Liebenzell!*

Doth it prey upon thy spirit,
Lack of ties thou lov'st so well?
Thy hall shall rival branch inherit?
*Send thy spouse to Liebenzell!*

Then sprightly son and rosy daughter,
Soon thy tide of hopes shall swell!
There's joy in store, while there is water,
*In the baths of Liebenzell!*

The Minstrel sung, the lady listened—
Then flew to try the magic spell:
Next Spring a son and heir was christened;
*Bless the baths of Liebenzell!*

---

